# New Mode of Stopping Hæmorrhage by Twisting the Mouths of the Vessels

**Published:** 1829-10-01

**Authors:** 


### XLVII.

### MODE OF STOPPING HÆMORRHAGE BY TWISTING THE MOUTHS OF THE VESSELS. By M. AMUSSAT.*

M. Amussat's encephalon must really be very prominent in the direction of the organ of *novelty*, if the professors of craniology allow us any such. One day we find him using none but straight sounds and catheters; on another we read of his treating varicocele by tying the spermatic arteries; and at present he has hit upon a plan whereby to supersede the necessity for securing blood-vessels with ligatures. If the worthy gentleman goes on in this career we shall soon have an entirely new system of surgery! We have noticed already the former improvements or discoveries of M. Amussat, and we are now in duty bound to do the same by the last. It is chiefly embodied in an oral communication made by the author to the Royal Academy of Medicine of Paris.

Reflecting on the well known fact that lacerated wounds are frequently not followed by hæmorrhage, M. Amussat conceived the idea, that methodically twisting arteries might perhaps be attended with the same result. A number of experiments were accordingly made on dogs, rabbits, horses, &c. and the following proceeding found to answer the purpose. An artery being cut across, the extremity is seized by means of a pair of forceps, the branches of which are closed by a spring. The end of the vessel is dragged out from the surface, for the distance of five or six lines, disengaged from the neighbouring parts, then seized with the thumb and fore-finger of the left hand, and twisted five or six times upon its axis by means of the forceps in the right. The twisting ought to be continued till the portion of the vessel in the bite of the instrument is broken. The end of the vessel now forms a cul-de-sac, which resists the impulse of the blood from behind, and is seen and felt to pulsate strongly. The twisting appears to act by rupturing the middle and internal coats, which instantly retire into the interior of the vessel and form a sort of plug or valve, whilst the vessel itself looks like a stump, enveloped and capped by the cellular membrane. If the vessel before being twisted is not seized with the left hand, it is injured up to the next collateral vessel. M. Amussat has tried the method twice on the human subject, once after extirpation of the testicle, and once after amputation, with success. Such is the substance of the first communication to the academy; in the second some points are touched on more at large.

* Journ. Hebdomadaire, Nos. 43-44.

In the first place, the effects of torsion or twisting are the same on arteries and veins; secondly, simple torsion, when employed to prevent hæmorrhage from arteries of considerable calibre, should consist of ten half turns; thirdly, that twenty half turns completely rupture the artery; fourthly, that after the complete or incomplete section of an artery the torsion should be made on each extremity; fifthly, that after a twisting methodically made we need never fear secondary hæmorrhage; sixthly, that torsion employed on the arteries of the dead human subject is attended with exactly the same effects, quoad the vessel, as in living animals, namely, the rupture of the inner coats and their retraction within the arterial tube, whilst the cellular membrane round them forms a kind of *capachon;* seventhly, that if the arteries are ossified, torsion breaks them; eighthly, that if we inject liquid into the twisted artery with whatever force we please, it does not escape by the twisted extremity, though sometimes the retracted inner tunics are driven out by the fluid, and the cellular membrane more or less distended; ninethly, that torsion has *all* the advantages of the ligature, *without* its inconveniences, and should instantly be adopted by army surgeons in particular.

In the discussion which ensued, the greater part of the speakers, among them M. Lisfranc and Baron Larrey, were opposed to M. Amussat's views. The opinions of Baron Larrey, in particular, are marked by good sense, and a rational estimation of the new proposal. The English reader cannot fail to perceive on how slender a foundation, practically speaking (two cases only) this plan for superseding the ligature has been built. It is true that experiments have been performed upon the lower animals, but, as M. Larrey justly remarked, such experiments, with regard to the properties of blood-vessels, are fallacious in the extreme. We all know how exceedingly difficult it is to make an aneurism by wounding the largest vessel in a brute, how readily the effect is produced in man. Were the torsion or twisting adopted in operations on the human subject, very probably the immediate bleeding would be controlled in a great many instances, but would not secondary hæmorrhage be more likely to succeed? We believe most firmly that it would, despite M. Amussat's dictum to the contrary. On the whole, it will require a vast deal more positive evidence than what is yet offered, to convince us even of the safety of M. Amussat's proposal.

---

